# Opposite-directional sex change in functional female protandrous anemonefish, *Amphiprion clarkii*: effect of aromatase inhibitor on the ovarian tissue

**DOI:** 10.1186/s40851-015-0027-y

**Published:** 2015-09-29

**Authors:** Masaru Nakamura, Saori Miura, Ryo Nozu, Yasuhisa Kobayashi

**Affiliations:** Sesoko Station, Tropical Biosphere Research Center, University of the Ryukyus, Sesoko 3422, Motobu, Okinawa 905-0227 Japan; Okinawa Churashima Foundation, 888 Ishikawa, Motobu, Okinawa 905-0206 Japan; Ushimado Marine Institute (UMI), Faculty of Science, Okayama University, Ushimado, Setouchi, 701-4303 Japan

**Keywords:** Anemonefish, Estrogen, Aromatase, Sex change, Protandrous hermaphrodite

## Abstract

**Introduction:**

The anemonefish, *Amphiprion clarkii*, is a protandrous hermaphrodite. Under appropriate social conditions, male fish can become female. Previous studies indicated that estrogens are important regulators of sex change in this fish. However, the mechanism of sexual plasticity in the gonad of this fish is still unknown. To elucidate the mechanisms underlying the sexual plasticity in the ovary of female anemonefish, an aromatase inhibitor (AI, 500 μg/g diet) was administered to the functional female fish for 80 days.

**Results:**

The levels of estradiol-17β (E2) in the fish treated with AI were significantly lower than those in the control group. Three out of five fish had ambisexual gonads with active spermatogenic germ cells in the ovarian tissue. However, female fish in the AI-treated group prior to treatment and those in the control group displayed no testicular characteristics in their developed ovaries. This result strongly suggests that germ cells with bipotentiality or spermatogonial cells remain in the functional ovaries of anemonefish following sex change from functional males to functional females. There is a possibility that estrogen depletion due to AI treatment might have caused the opposite-directional sex change from functional female to male in the anemonefish.

**Conclusions:**

The anemonefish keeps their high sexual bipotential in the ovary after sex change.

## Introduction

Sex change is a common phenomenon among marine fishes, especially in the tropical and sub-tropical regions [1–3]. Despite the many ecological and physiological studies of sex change in protandrous anemonefishes, the physiological events that trigger natural sex change have not yet been elucidated in detail [4–8].

Anemonefishes (genus *Amphiprion*) have a monogamous mating system, which is maintained by protandrous sex change [9–11]. During the male phase, the gonads consist of both ovarian tissue with immature oocytes and testicular tissue with active spermatogenesis. In contrast, during the functional female phase, only the ovarian tissue is present [5, 6, 9, 10]. We are particularly interested in the destiny of the spermatogenic germ cells, specifically the spermatogonial cells, which have the ability to differentiate into oocytes or sperm, following changes in the ovaries of functional females. We recently discovered that the ovaries of adult gonochoristic fishes, such as Medaka (*Oryzias latipes*), Nile tilapia (*Oreochromis niloticus*) and zebrafish (*Danio rerio*) had the ability to change into testes with active spermatogenic germ cells following estrogen depletion by treatment with aromatase inhibitors [12, 13]. However, the molecular mechanisms that dictate whether the ovarian cells in anemonefish differentiate into oogonia or spermatogonia after natural sex change remain unclear.

Although the exact endocrine mechanism involved in the sex change in hermaphrodite fish is unclear, sex steroids are believed to regulate the process [14, 15]. The change from female to male is accompanied by a decrease in the plasma estradiol-17ß (estrogen; E2) levels and a gradual increase in plasma 11-ketotestosterone (androgen; 11-KT) levels in some protogynous fishes [16–19]. In the case of protandrous anemonefish, *in vivo* and *in vitro* studies show that 11-KT levels are high and E2 levels are low in the functional males, whereas 11-KT levels are low and E2 levels are high in the functional females [4, 6, 8]. It is also noteworthy that administration of E2 to anemonefish with ambisexual gonads led to the degradation and disappearance of testicular tissue in the gonads [20]. These findings strongly suggest that E2 plays an important role in gonadal formation and sex change in protandrous hermaphrodite fishes.

In the present study, to clarify the role of estrogen in anemonefish sex changes and to elucidate the mechanism of sexual plasticity in the germ cells within the functional ovaries of these protandrous fish, we examined the effects of AI on ovaries during the female phase.

## Materials and methods

### Experimental animals

Wild-type, sexually mature female anemonefish (*n* = 15; Total length: 11.56 ± 0.35 cm, Standard length: 9.15 ± 0.24 cm, Body weight: 38.38 ± 3.15 g) were purchased from a fisherman in Nakijin, northern Okinawa, Japan, on 17 May 2007.

### Aromatase inhibitor treatment

The appropriate amount of AI (Fadrozole; Novartis Pharma Inc., Tokyo, Japan) was dissolved in ethanol and mixed into commercial fish food (Otohime B2: Nisshin Seifun, Tokyo, Japan). The food was dried overnight at 37 °C before use. Only ethanol was added in the food for the control groups. We sacrificed 5 females as an initial control group before AI treatment. The fish were divided into two groups: AI-treated group and untreated control group. The fish were kept in glass tanks (60 cm × 30 cm × 36 cm) and reared in a flow-through system supplied with seawater. The AI was added at a dose of 500 μg/g of food. The fish were fed enough once daily for 80 days.

### Sampling and histological analysis

After anesthetization with 2-phenoxyethanol, the total length, the standard length and the body weight of each fish were measured. Blood was collected in a heparinized syringe, centrifuged at 15,000 rpm for 10 min, and the collected plasma was stored at −30 °C until analysis. The gonads were removed and weighed to calculate gonadosomatic index (GSI = gonad weight / body weight × 100). The gonads were fixed overnight at room temperature in Bouin’s solution and then dehydrated in alcohol, clarified in benzene, and embedded in paraffin. The tissues were cut into 7-μm thick cross-sections, and the sections were stained with hematoxylin and eosin following conventional histological procedures. All animal handling and experiments were conducted in accordance with our Guide for Care and Use of Laboratory Animals (Doubutu-jikken-kisoku, 19.6.26) approved by the University of the Ryukyus.

### Enzyme-Linked Immunosorbent Assay (ELISA)

The concentrations of 11-KT and E2 in plasma were determined by ELISA following routine procedures [21]. The 11-KT anti-serum had a cross-reactivity of less than 2.7 % and less than 0.2 % against testosterone (T) and estradiol-17β (E2), respectively. The E2 anti-serum had a cross-reactivity of less than 0.05 % against testosterone (T) and no cross-reactivity against 11-KT.

### Statistics

The data on GSI and plasma steroid hormones were expressed as the mean ± S.E.M. (standard error of mean). For GSI, arc sin-transformation of the square root was applied prior to subsequent statistical analysis. The values of GSI and plasma hormones between experimental groups were analyzed by one-way analysis of variance (ANOVA). Significant effects were further analyzed using Fisher’s PLSD post-hoc test. Statistical analyses were performed in Stat View 4.5 J (SAS Institute, Inc., Cary, NC). All tests were two tailed, and a P value < 0.05 was considered significant.

## Results

**Fig. 1:**
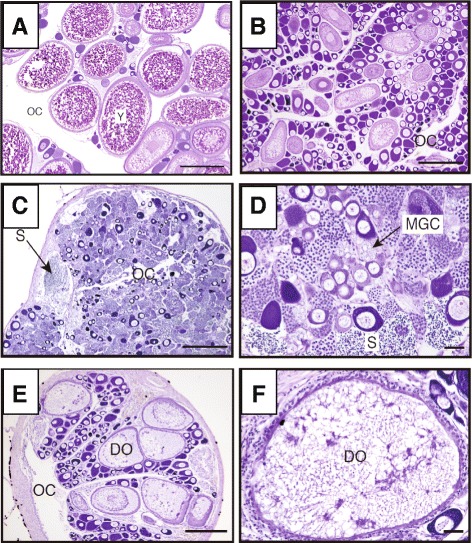
Histological images of gonads in anemonefish *Amphiprion clarkii.* The gonads of fish in the initial control group (**a**). The gonads of fish in the control group (**b**), the ambisexual gonads and the ovary of fish in the Aromatase inhibitor (AI)-treated group (**c, d, e** and **f**). OC, ovarian cavity; DO, degenerating oocyte; MGC, meiotic germ cells; Y, yolky oocyte. S, sperm. Scale bars = 200 μm in (**a, b, c** and **e**) Scale bar = 20 μm in (**d** and **f**)

The mean GSI in the control group (4.14 ± 0.09) was slightly higher than that in the AI administration group (3.08 ± 0.30). All of the fish in the initial control and control groups possessed mature ovaries that were characterized by many yolky oocytes and no discernible testicular tissue (Fig. 1a, b). However, in the AI-treated group, three out of five fish had ambisexual gonads displaying both testicular tissue, including cysts of active spermatogenic cells, and ovarian tissue, including many meiotic oocytes and oocytes in the peri-nucleolus stage (Fig. 1c, d). Although there was no testicular tissue in the gonads of the remaining two fish, there were many degenerating oocytes and hypertrophied granulosa cells mixed among young oocytes at the peri-nucleolus stage and cysts of meiotic oocytes (Fig. 1e, f).

**Fig. 2:**
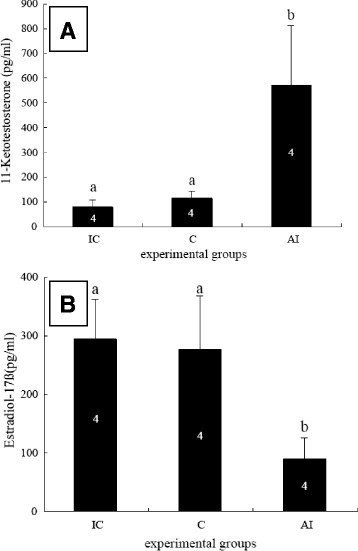
Plasma levels of estradiol-17β (**a**) and 11-ketotestosterone (**b**) in experimental and control groups presented as the mean ± S.E.M. Numbers in columns were sample size. Different superscript letters (**a, b**) indicate significant differences between phases at *P* < 0.05 (Fisher’s PLSD post-hoc test). IC, initial control; C, control group; AI, the aromatase inhibitor-treated group

The mean plasma 11-KT level in the AI-treated group (569.3 ± 242.4 pg/ml) was significantly higher than that in the initial control group (77.3 ± 28.0 pg/ml) and the control group (114.4 ± 27.2 pg/ml) (Fig. 2a). The mean plasma E2 level in the AI-treated group (87.8 ± 35.6 pg/ml) was significantly lower than that in the initial control group (291.5 ± 68.1 pg/ml) and the control group (273.9 ± 77.0 pg/ml) (Fig. 2b).

## Discussion

It is known that the gonads in functional female anemonefishes consist of only ovarian tissue and no testicular tissue [6, 9, 10]. In the present study, we confirmed that the females in the initial control group were indeed functional and that their gonads were comprised of mature ovarian tissue, completely devoid of testicular tissue. However, following treatment with the aromatase inhibitor, Fadrozole, three out of five functional female fish presented with ambisexual gonads, which possessed active spermatogenic tissues including a large amount of sperm similar to what is seen in functional males, while the gonads from untreated females retained developed ovaries with no testicular tissue. We also observed that the E2 levels in fish treated with AI were significantly lower than that in the control group. These results strongly suggest that inhibition of E2 synthesis by AI treatment induces mature testicular tissue formation in functional ovaries, a finding that has never been reported in the protandrous anemonefish.

The exact origin of the spermatogenic cells and testicular somatic cells in the female gonads remains unknown because there is no apparent testicular tissue in the ovary [22]. Previously, we induced female to male sex changes by AI treatment in some protogynous fish, namely, three-spot wrasse (*Halichoeres trimaculatus*) [18], and grouper (*Epinephelus merra*) [23, 24]. It was suggested that some types of ovarian somatic cells survived and that the gonial germ cells spreading across the ovary may have differentiated into sperm during the gonadal sex changes in three-spot wrasse [22]. This study implied that the origin of testicular cells during sex changes is the ovarian cells forming the ovarian tissue [22]. In addition, we induced sex change from adult females to mature males in gonochoristic Nile-tilapia (*Oreochromis niloticus*)*,* Medaka (*Oryzias latipes*)*, and* zebrafish (*Danio rerio*) [12, 13]. These results revealed that following sex differentiation, germ cells and somatic cells in the ovary retain bipotency for long time in the ovary of gonochoristic fish. In addition, previous reports involving rainbow trout (*Oncorhynchus mykiss*) show that testicular germ cells possess a high level of developmental plasticity and sexual bipotency, even after the animal reaches maturity [25]. Based on these findings, we conclude that some germ and somatic cells in the ovaries of functional female protandrous anemonefish retain bipotency, which enables them to re-differentiate into testicular tissue.

We found that mean plasma 11-KT levels significantly increased following AI treatment, while mean plasma E2 levels were significantly lowered in the same treatment group. This same decrease in the E2 plasma level was observed during female to male sex changes in protogynous fishes; however, the increase in plasma 11-KT levels was less universal [16–19], indicating that the decrease in estrogen levels may be more important to the sex change than the increase of androgen. In addition, E2 compensation during AI treatment suppressed the sex change in three-spot wrasse fish [18]. Additionally, treatment of ambisexual gonads with E2 after testicular differentiation in anemonefish causes the disappearance of testicular tissue [20]. These results indicate that estrogen has an important role in ovarian differentiation and in sex change in hermaphrodite fish.

Moreover, E2 treatments around the time of testicular differentiation suppressed the differentiation of testicular tissue [26, 27]. In the protandrous black porgy, *Acanthopagrus schlegeli*, oral administration of AI suppressed aromatase activity and inhibited the natural sex change from male to female [14, 28–30]. Furthermore, we found that inhibition of E2 synthesis with AI was shown to induce testicular differentiation in the developed ovaries of anemonefish, which agrees with previous reports showing that high E2 levels restrict the ovarian transition to testis in the black porgy. Thus, it is possible that high levels of estrogen act to maintain ovarian tissue, whereas extremely low levels of estrogen enable testicular differentiation. Therefore, there is a high possibility that some gonial germ cells and somatic cells in the ovaries differentiate into testicular tissue under conditions of E2 depletion.
